# Circulating Progranulin: A Promising Novel Diagnostic and Prognostic Biomarker in Canine Oncology

**DOI:** 10.3390/ani15111605

**Published:** 2025-05-30

**Authors:** Keon Kim, Yeong Jun Kim, Chang Hyeon Choi, Yoon Jung Do, Woong Bin Ro, Chang Min Lee

**Affiliations:** 1 Department of Veterinary Internal Medicine, College of Veterinary Medicine, Chonnam National University, Gwangju 61186, Republic of Korea; 2 Department of Veterinary Emergency and Critical Care Medicine, College of Veterinary Medicine, Chonnam National University, Gwangju 61186, Republic of Korea; 3 College of Veterinary Medicine and BK21 FOUR Program, Chonnam National University, Gwangju 61186, Republic of Korea; 4 Department of Laboratory Animal Medicine, College of Veterinary Medicine, Chonnam National University, Gwangju 61186, Republic of Korea; 5 Division of Animal Diseases & Health, National Institute of Animal Science, Rural Development Administration, Wanju-gun 55365, Republic of Korea

**Keywords:** diagnosis, dog, GP88, prognosis, serum, tumor

## Abstract

Progranulin (PGRN) is a pluripotent growth factor that has shown promise as a diagnostic and prognostic biomarker for various neoplastic conditions in humans. This study aims to explore the PGRN as a novel biomarker for diagnosing and predicting the prognosis in canine tumors. Dogs (*n* = 104) with tumors as the chief complaint were selected and classified based on clinical categorization, malignancy, and metastasis. The control group (*n* = 30) consisted of healthy dogs with no evidence of neoplastic diseases. Serum PGRN levels were quantified using enzyme-linked immunosorbent assay (ELISA). Dogs with tumors exhibited significantly elevated PGRN levels compared to control dogs (*p* < 0.0001), with a high sensitivity of 90.91%. Malignant tumors demonstrated markedly higher PGRN levels relative to the control group (*p* = 0.0012), while no significant difference was found between benign tumors and the control group. Additionally, serum PGRN was identified as a significant marker for differentiating metastatic tumors from non-metastatic ones (*p* = 0.0264). PGRN exhibited high sensitivity for tumor detection, suggesting that it may serve as a screening biomarker. Prognostically, increased PGRN correlated with unfavorable outcomes, notably linked to malignancy and metastasis. This study underscores the potential of PGRN as a novel biomarker with early diagnostic and prognostic value in canine oncology.

## Simple Summary

Progranulin (PGRN) is a versatile growth factor involved in numerous physiological processes, and its dysregulation has been implicated in the development of various diseases. Notably, its overexpression has been identified in human cancers, where it significantly contributes to tumor progression. However, until now, no veterinary studies have explored the clinical value of measuring serum PGRN levels in dogs diagnosed with tumors. This study suggests that PGRN is promising both as an early diagnostic and a prognostic biomarker for canine tumors. PGRN exhibited high sensitivity for tumor detection, indicating that it may have potential as an effective screening biomarker. Additionally, it proved valuable in distinguishing between metastatic and non-metastatic tumors. Prognostically, increased levels of PGRN correlated with unfavorable outcomes, notably linked to malignancy and metastasis. These results highlight PGRN’s potential as an important biomarker for diagnostic and prognostic evaluation in canine oncology.

## 1. Introduction

Tumors are among the leading causes of mortality in companion dogs. Although earlier reports estimated approximately 5300 cases per 100,000 dogs annually [1], more recent canine cancer registries in Europe report incidence rates ranging from 775 to 1701 tumors per 100,000 dog-years [2,3]. Despite differences in reporting methods, the incidence of canine tumors remains significantly higher than that observed in humans, underscoring the importance of early diagnosis and accurate prognostic determination. Histopathological examination is generally required for the definitive diagnosis as a gold standard. Furthermore, cytologic evaluation often provides a rapid, cost-effective, and reliable means of distinguishing between benign and malignant lesions. Nevertheless, in veterinary clinical fields, the cost burden, invasiveness, or owner reluctance to pursue such diagnostics may delay diagnosis and hinder timely interventions. In such scenarios, minimally invasive screening methods can play a pivotal role, with serum-derived circulating biomarkers emerging as a particularly valuable tool. These biomarkers offer high accessibility and utility as monitoring indicators, facilitating longitudinal assessments and enabling effective prognostication. Indeed, several studies have reported attempts to utilize serum-derived biomarkers for the diagnosis of malignant tumors in dogs [4,5], and one of these has even been commercialized as a diagnostic kit [6].

Progranulin (PGRN), also referred to as 88-kDa glycoprotein (GP88), acrogranin, or proepithelin, is a pluripotent growth factor that plays a critical role in various physiological functions [7,8]. In embryonic development, PGRN is closely associated with developmental processes and placental formation. In adults, it is involved in a wide array of regulatory mechanisms, including tissue regeneration, angiogenesis, inflammation, bone and cartilage development, immune response modulation, and neuroprotection [7]. The dysregulation of PGRN has been implicated in the pathogenesis of various diseases, with its overexpression in human cancers recognized as a key driver of tumor progression [8]. Consequently, PGRN has been established as a diagnostic biomarker in several neoplastic diseases, and studies have demonstrated correlations between serum PGRN levels and cancer prognosis, as well as recurrence in certain cancers [9,10,11,12,13]. Notably, PGRN has been consistently detected not only in neoplastic tissues but also in urine and blood from cancer patients, further emphasizing its potential as a non-invasive biomarker for cancer diagnostics [14,15,16].

To date, serum-derived biomarkers for the early diagnosis and prognostic evaluation of tumors have not been clinically established in the veterinary field, and most definitive diagnoses require invasive methods. Furthermore, research on non-invasive, blood-based markers for the clinical management and prognostic monitoring of canine tumors has been very limited. This poses a limitation in veterinary clinical practice regarding the early detection of tumors and the determination of an appropriate timing for therapeutic intervention. Therefore, this study highlights the need for tumor-associated biomarkers that are measurable in canine serum. It evaluated whether the clinical value of PGRN, whose utility has been confirmed in humans, could also be established in canine tumors. Through this preliminary study, we aim to explore the potential of serum-derived PGRN as a valid biomarker for diagnosing and assessing the prognosis of canine tumors.

## 2. Materials and Methods

### 2.1. Animals

The tumor group (*n* = 104) consisted of dogs that were retrospectively selected based on their initial presentation for neoplastic diseases as the primary complaint, with no prior history of tumor treatment. These cases were enrolled from the Chonnam National University veterinary teaching hospital and local animal hospitals from June 2021 to December 2024. The inclusion criteria required dogs with tumors identified by physical examination and diagnostic imaging to undergo histopathological examination for enrollment in the tumor group. Histopathological data for all included cases were retrospectively collected through a medical record review, except for lymphoma. Only the definitive diagnosis of lymphoma was established based on the cytologic examination of fine-needle aspiration (FNA) samples, with confirmation using the Polymerase Chain Reaction for Antigen Receptor Rearrangement (PARR) analysis. For further analysis, dogs with tumors were classified based on clinical categorization, malignancy, and the presence of metastasis. Specifically, the clinical categorization and malignancy of tumors were determined according to the results of histopathological examination. Practical clinical categorization was performed using a classification system that is widely utilized in small animal clinical practice [17]. Both regional and distant metastases were investigated and confirmed through physical examination, radiography, ultrasonography, and computed tomography (CT) imaging along with FNA methods. In the tumors with metastasis group, cases were further categorized based on the type of metastasis.

The control group (*n* = 30) consisted of healthy dogs presenting for a health checkup at the CNU veterinary teaching hospital and local animal hospitals. These dogs were evaluated based on their medical history, physical examinations, blood tests, and imaging studies (radiography and abdominal ultrasonography).

In both groups, dogs with recent surgical history, musculoskeletal disorders, severe inflammatory diseases or other conditions that are known to be able to influence PGRN concentrations were excluded. Additionally, dogs on medication due to underlying conditions were also excluded. The experimental design was approved by the Chonnam National University Institutional Animal Care and Use Committee (CNU IACUC-YB-2021-70 and CNU IACUC-YB-2024-78).

### 2.2. Serum Sampling

Using surplus serum from dogs with neoplastic diseases and healthy dogs, PGRN concentrations were measured. All serum samples were obtained by centrifugation at 4000 RPM for 10 min within 1 h after blood collection, and stored at −20 °C until the measurement of PGRN.

### 2.3. Enzyme-Linked Immunosorbent Assay (ELISA)

Levels of circulating PGRN were measured within the IACUC approval period using commercial enzyme-linked immunosorbent assay (ELISA) kits for PGRN (Canine Progranulin ELISA Kit, MyBioSource Inc., San Diego, CA, USA), following the manufacturer’s protocols. In brief, 96-well plates were incubated with standards and horseradish peroxidase (HRP)-conjugate reagent, and separately, serum samples were also incubated with HRP-conjugate reagent for 1 h at 37 °C. After several aspiration/wash processes, chromogen solution was added to each well of the plates for 15 min at 37 °C and protected from light. The substrate reaction was terminated by the addition of a stop solution prepared with 2N sulfuric acid (H_2_SO_4_). Optical density was measured at 450 nm. Protein levels were calculated according to standard curves. The kit manufacturer indicates that the intra- and inter-assay coefficients of variation were <15% for the Progranulin ELISA kit (MyBioSource Inc., San Diego, CA, USA), respectively.

### 2.4. Statistical Analyses

All statistical data were analyzed using commercially available software (GraphPad Prism v10.2, GraphPad Software Inc., La Jolla, CA, USA). The choice of statistical tests was based on the distribution characteristics and the structure of the data. The Shapiro–Wilk test was performed to determine normality. Based on the distribution characteristics, a parametric Student’s t test was performed to confirm the significance of PGRN between the tumor group and the control group. Similarly, considering the distribution characteristics, PGRN levels according to different tumor origins and malignancy statuses were evaluated using the Kruskal–Wallis test followed by Dunn’s post hoc analysis for non-parametric data. To compare PGRN concentrations based on the presence or absence of metastasis, a one-way analysis of variance (ANOVA) with Tukey’s post hoc test was performed. Receiver operating characteristic (ROC) analyses were used to confirm the diagnostic accuracy and compare the diagnostic sensitivity and specificity. Statistical significance was set at *p* < 0.05 for all analyses.

## 3. Results

### 3.1. Caseload

A total of 110 dogs with tumors were retrospectively selected as the tumor group. An additional comparative analysis among groups was performed on 104 cases, excluding four cases without histopathological examination and two cases with more than two types of tumors. The descriptive characteristics of dogs with tumors and controls that were included in the study are presented in Table 1. Additionally, the definitive diagnosis in 104 dogs with tumors are summarized in Table 2. In the mammary gland tumor (MGT) cases, it is challenging to specify a particular histogenesis due to the potential for multiple occurrences across different mammary glands and the varying origins of each mammary gland. Accordingly, the histopathological diagnosis of the most representative tumor is documented in Table 2. In the 11 cases of MGT, although surgical excision followed by histopathological evaluation had been conducted, the corresponding data were missing in the retrospective records and therefore classified as “unavailable”. 

To confirm demographic equivalence among the groups, statistical comparisons were performed. As shown in Table 1, age differences among groups were assessed using the Kruskal–Wallis test followed by post hoc analysis. A statistically significant difference in age was observed only between the control and MGT groups (*p* = 0.0294), while no significant differences were identified between the control group and the other tumor groups. Group differences based on sex were evaluated using Fisher’s exact test. A significant sex distribution difference was found exclusively in the MGT group compared to the control group (*p* < 0.0001), which aligns with the known predisposition of MGTs to occur in female dogs. With respect to neuter status, Fisher’s exact test revealed significant differences between the control and MGT groups (*p* < 0.0001), control and HLS groups (*p* = 0.0046), and control and epithelial tumor groups (*p* = 0.0269). Regarding breed characteristics, a comparison based on purebred status using Fisher’s exact test showed a significant difference only between the control and HLS groups (*p* = 0.0328).

### 3.2. Serum PGRN Concentrations in Tumor and Control Groups

**Figure 1:**
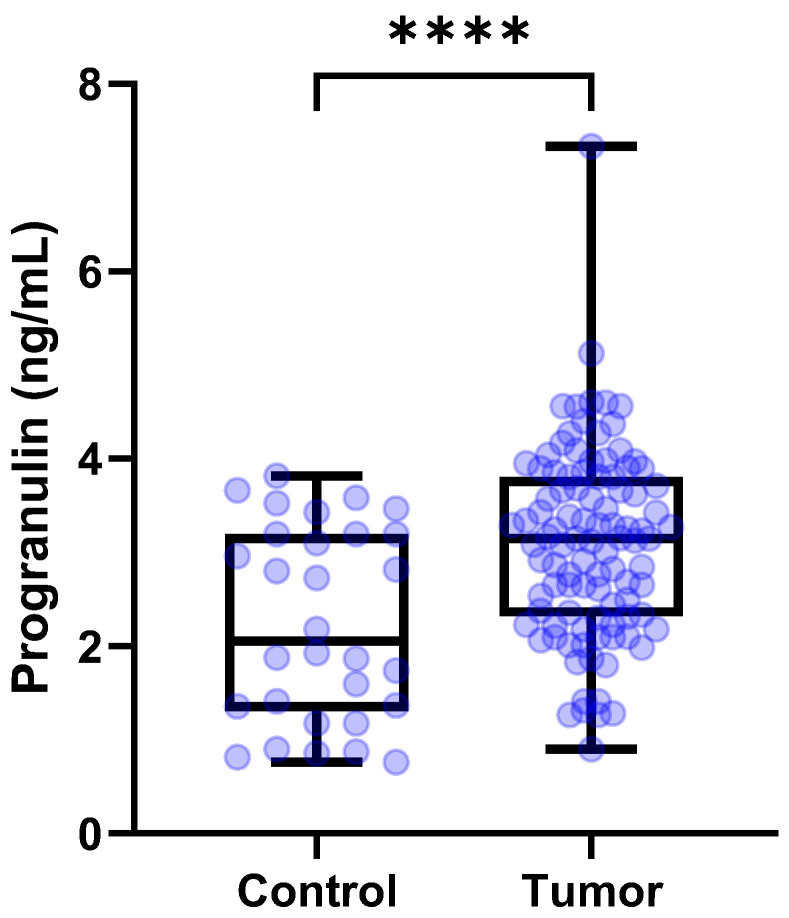
**Box plots of PGRN concentrations in dogs with tumors and control group.** Dogs with tumors showed significantly higher PGRN concentrations than control group (*p* < 0.0001). The student’s *t* test. **** *p* < 0 .0001. PGRN, progranulin.

The concentrations (median [IQR]) of serum PGRN in control dogs (*n* = 30) were 2.054 [1.312–3.194] ng/mL. The median [IQR] circulating serum PGRN levels in dogs with tumors (*n* = 110) were 3.154 [2.330–3.853] ng/mL. Circulating PGRN levels were significantly higher in the dogs with tumors than in the control dogs (*p* < 0.0001) (Figure 1).

### 3.3. Comparison of PGRN Levels Among Tumor Types Based on Tissue or Cell Origin

**Figure 2:**
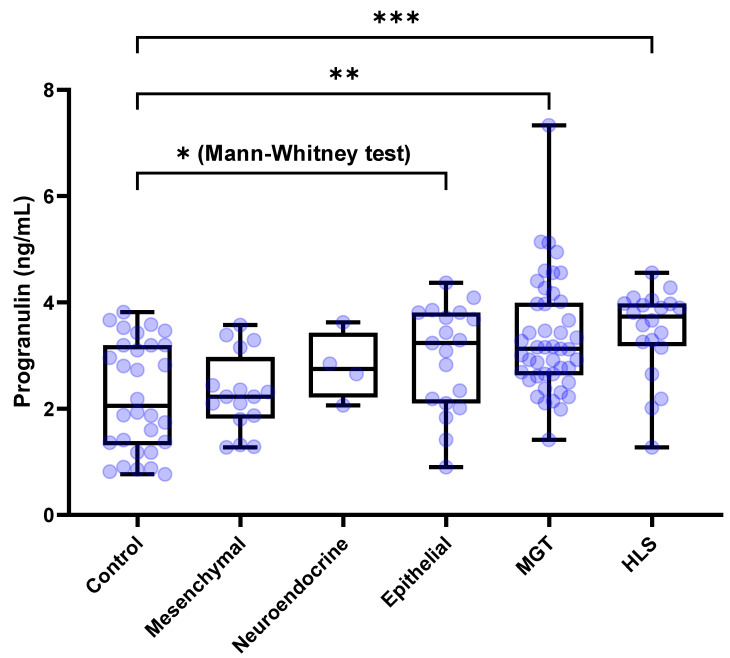
**Box plots of PGRN concentration for dogs with tumors and the control group based on the tissue or cell origin.** Dogs with MGTs and HLS-derived tumors showed significantly higher PGRN concentrations than the control dogs (*p* = 0.0027 and *p* = 0.0007, respectively). However, there were no significant differences of PGRN concentrations for dogs with mesenchymal, epithelial, and neuroendocrine tumors compared to the control dogs. However, the Mann–Whitney test showed significantly higher PGRN level for epithelial tumors than that of the control group (*p* = 0.0118). Kruskal–Wallis test followed by Dunn’s post hoc analysis and Mann-Whitney test. * *p* < 0.05; ** *p* < 0.01; *** *p* < 0.001. PGRN, progranulin; MGT, mammary gland tumor; HLS, hematopoietic and lymphoreticular system.

The serum concentration of PGRN for healthy control dogs and with (*n* = 30), MGTs (*n* = 45), hematopoietic and lymphoreticular system (HLS) tumors (*n* = 20), mesenchymal tumors (*n* = 16), epithelial tumors (*n* = 19), and neuroendocrine tumors (*n* = 4) were 2.054 [1.312–3.194], 3.125 [2.632–3.991], 3.733 [3.175–3.979], 2.226 [1.816–2.971], 3.232 [2.098–3.806] and 2.748 [2.211–3.427] ng/mL, respectively. The Kruskal–Wallis test demonstrated significant differences among the groups (*p* < 0.0001). Dunn’s post hoc test showed that the circulating PGRN levels were significantly higher in dogs with MGTs and HLS tumors than in the control dogs (*p* = 0.0027 and *p* = 0.0007, respectively) (Figure 2). By contrast, serum PGRN levels did not show the difference in dogs with epithelial (*p* = 0.3599), mesenchymal (*p* > 0.9999), and neuroendocrine tumors (*p* > 0.9999) compared to the control dogs. However, the Mann–Whitney test showed significantly higher PGRN level for epithelial tumors than that of the control group (*p* = 0.0118).

### 3.4. Comparison of PGRN Levels According to the Malignancy of Tumors

**Figure 3:**
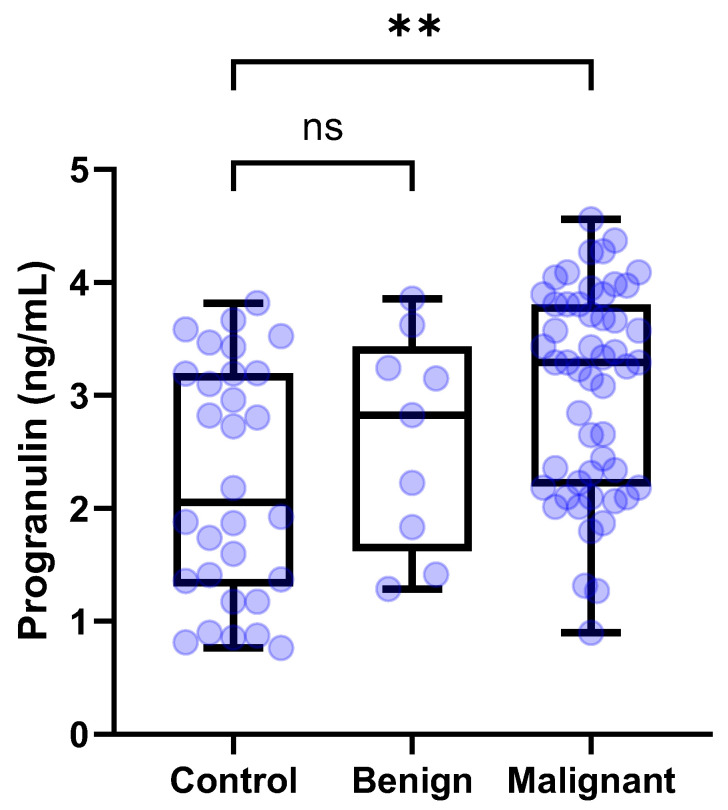
**Box plots of PGRN concentration in dogs with tumors and control dogs according to malignancy.** Dogs with malignant tumors showed significantly higher PGRN concentrations than control dogs (*p* = 0.0012). By contrast, dogs with benign tumors showed no significant difference of PGRN concentrations compared to control dogs. Kruskal–Wallis test followed by Dunn’s post hoc analysis. ** *p* < 0.01; ns, no significance. PGRN, progranulin.

The median [IQR] circulating PGRN levels were compared among the control dogs (*n* = 30), dogs with benign tumors (*n* = 9), and dogs with malignant tumors (*n* = 52). The serum concentrations of PGRN for the control dogs, dogs with benign tumors, and dog with malignant tumors were 2.054 [1.312–3.194], 2.825 [1.623–3.432], and 3.289 [2.194–3.806] ng/mL, respectively. The Kruskal–Wallis test confirmed significant differences among the groups (*p* = 0.0017). According to the post hoc tests, circulating PGRN levels were significantly higher in dogs with malignant tumors than in the control dogs (*p* = 0.012) (Figure 3). However, the serum PGRN concentrations in dogs with benign tumors were not significantly different from those in the control group (*p* > 0.9999).

### 3.5. Comparison of PGRN Levels Based on the Presence of Tumor Metastasis

**Figure 4:**
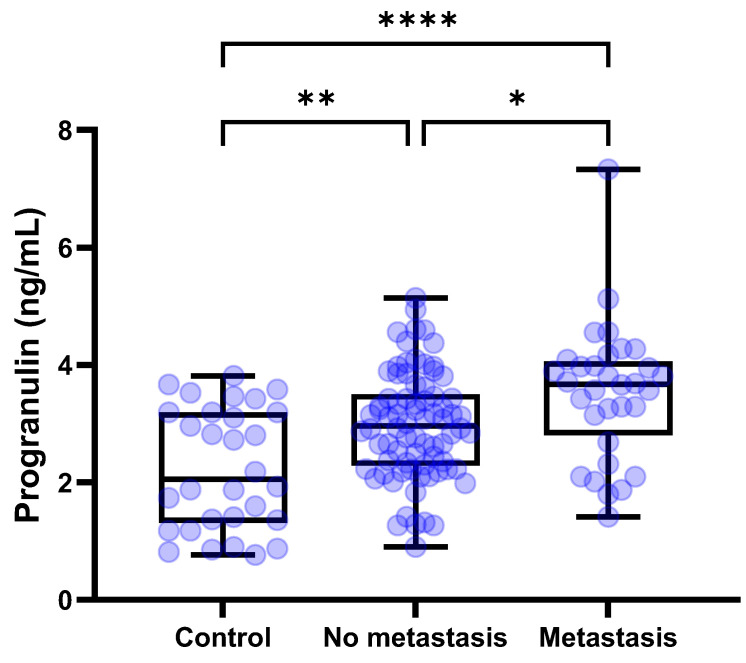
**Box plots of PGRN concentration in dogs with tumors and control dogs based on metastasis.** Dogs with tumors showed significantly higher PGRN concentrations than control dogs regardless of the metastasis (*p* < 0.0001 and *p* = 0.0024, respectively). Furthermore, in dogs with metastatic tumors, PGRN levels were significantly higher compared to tumors without metastasis (*p* = 0.0264). One–way ANOVA test followed by Tukey’s post hoc analysis. * *p* < 0.05; ** *p* < 0.01; **** *p* < 0.0001. PGRN, progranulin.

The median [IQR] circulating PGRN levels were compared among the control (*n* = 30), tumors without metastasis (*n* = 78), and tumors with metastasis (*n* = 32). Serum concentrations of PGRN for the control group, tumors without metastasis, and tumors with metastasis were 2.054 [1.312–3.194], 2.964 [2.283–3.505], and 3.671 [2.802–4.062] ng/mL, respectively. The one-way ANOVA test demonstrated significant differences among the groups (*p* < 0.0001). According to the post hoc tests using Tukey’s method, circulating PGRN levels were significantly higher in both tumors with metastasis and without metastasis than in the control (*p* < 0.0001 and *p* = 0.0024, respectively) (Figure 4). Furthermore, serum PGRN concentrations in tumors with metastasis demonstrated a significant difference compared to the tumors without metastasis (*p* = 0.0264). The tumors with metastasis group (*n* = 32) was subdivided into regional metastasis (*n* = 19), distant metastasis (*n* = 9), and both regional and distant metastasis (*n* = 4) subgroups. The serum concentrations of PGRN for the regional metastasis, distant metastasis, and both metastasis subgroups were 3.571 [2.312–3.972], 3.571 [2.509–3.759], and 4.840 [2.199–6.799] ng/mL, respectively. No statistically significant differences were observed among the subgroups.

### 3.6. Comparison of Diagnostic Accuracy Through Receiver Operating Characteristic (ROC) Curve Analyses

**Figure 5:**
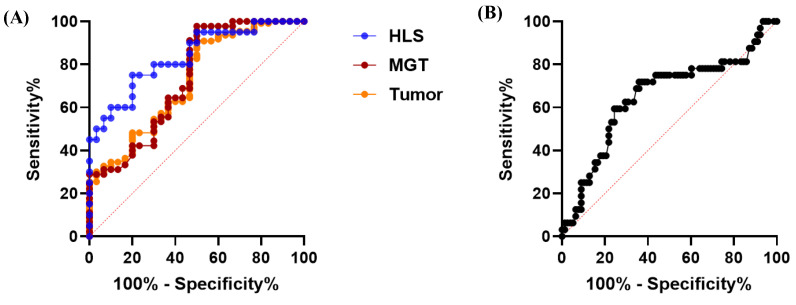
**ROC curve of PGRN concentrations to confirm the diagnostic and prognostic accuracy of tumors.** (**A**) ROC curve for predicting the dogs with tumors (AUC = 0.723; *p* = 0.0002), MGTs (AUC = 0.734; *p* = 0.0006), and HLS tumors (AUC = 0.835; *p* < 0.0001) compared to healthy dogs. (**B**) ROC curve for predicting the metastasis of canine tumors (AUC = 0.651; *p* = 0.0129) compared to the canine tumors without metastasis. ROC, receiver operating characteristic; PGRN, progranulin; AUC, area under the curve; MGT, mammary gland tumor; HLS, hematopoietic and lymphoreticular system.

To evaluate the potential of PGRN as a diagnostic biomarker for tumors, receiver operating characteristic (ROC) analyses were conducted in control and dogs with tumors. The analysis indicated that serum PGRN level could be used to differentiate dogs with tumors, MGTs, and HLS tumors from healthy dogs, with an ROC area under curve (AUC) of 0.7229 (95% confidence interval [95% CI], 0.6182–0.8275; *p* = 0.0002), 0.7337 (95% CI, 0.6142–0.8532; *p* = 0.0006), and 0.835 (95% CI, 0.7204–0.9496; *p* < 0.0001) (Figure 5A). Moreover, the PGRN level could be used to differentiate tumors with metastasis from those without metastasis, with an ROC AUC of 0.6514 (95% CI, 0.5311–0.7718; *p* = 0.0129) (Figure 5B). The most appropriate cut-off values with sensitivities and specificities obtained from the ROC analyses are presented in Table 3.

## 4. Discussion

In this study, we evaluated and comparatively analyzed the concentration of PGRN in dogs with tumors and controls. The results revealed a significantly elevated level of PGRN in dogs with tumors compared to the control group (*p* < 0.0001). ROC analysis for the tumor differentiation demonstrated high sensitivity (90.91%). Furthermore, serum PGRN was found to be a significant marker in distinguishing metastatic from non-metastatic tumors (*p* = 0.0264), suggesting that it may have potential utility as a prognostic biomarker for tumor-related diseases. In addition, an assessment of PGRN levels based on tumor malignancy revealed that malignant tumors exhibited significantly higher PGRN levels compared to the control group (*p* = 0.0012). By contrast, benign tumors did not show a significant difference from the controls. Moreover, metastatic tumors demonstrated substantially elevated PGRN levels relative to the control group (*p* < 0.0001). These findings suggest a potential association between elevated PGRN levels and poor prognostic factors, such as tumor malignancy and metastasis.

Growth factor PGRN is known to regulate tumorigenesis in various types of cancers by stimulating cell proliferation, migration, invasion, angiogenesis, malignant transformation, drug resistance, and immune evasion [18]. In this study, serum PGRN levels in dogs with tumors were significantly higher compared to the healthy control group. Upon practical clinical categorization of tumors, the Kruskal–Wallis test and post-hoc analysis revealed significantly elevated levels of PGRN in HLS tumors and MGT compared to the controls. Additionally, although epithelial tumors did not initially show statistical significance in the Kruskal–Wallis test, Mann–Whitney testing confirmed significantly higher PGRN levels in epithelial tumors compared to the controls. These findings align closely with previous human studies involving carcinoma of breast cancer [19], ovarian cancer [20], lymphocytic leukemia [21,22], and lymphoma [23], suggesting that PGRN levels may be elevated in various types of tumors, rather than being specific to individual tumor types. This result also supports previous research suggesting that PGRN could act as an essential molecule in tumorigenesis through chronic inflammation or interactions within the tumor microenvironment, regardless of target organs or tissues [23]. Therefore, serum PGRN could serve as a relatively broad-spectrum tumor biomarker in dogs, and its application may be particularly advantageous in MGTs, HLS tumors, and epithelial tumors rather than mesenchymal tumors.

The PGRN levels exhibited a significant difference between dogs with tumors and the control group (3.154 vs. 2.054 ng/mL, *p* < 0.0001). Notably, ROC analysis revealed a high sensitivity (90.91%) at a cut-off value of 1.956 ng/mL, suggesting its potential utility as an early diagnostic biomarker. This finding indicates that serum PGRN may be useful for screening purposes in canine tumor diagnosis, where serum PGRN levels below 1.956 ng/mL suggest a very low likelihood of tumor presence. Ideally, screening biomarkers used to diagnose occult cancers should have a high sensitivity of 90% or more [24]. The results of this study demonstrate that PGRN achieved a sensitivity of over 90% at its optimal cutoff value, supporting its potential as a screening tool. Tumors often present without clinical signs, and when clinical signs do manifest, the disease is frequently already at an advanced stage. Therefore, early diagnosis is crucial, and PGRN may hold promise as an early diagnostic biomarker for canine tumors.

In this study, a significant difference in serum PGRN concentrations was observed between malignant tumors and the control group (3.289 vs. 2.054 ng/mL, *p* = 0.0012), while no significant difference was found between benign tumors and the control group. This finding suggests that elevated PGRN levels may indicate the likelihood of malignancy in dogs. These results align with previous research demonstrating that PGRN contributes to the development of malignant tumors in vitro [25,26,27]. Consequently, measuring serum PGRN levels in clinically healthy dogs without visible masses can be helpful as an indicator to recommend further diagnostic evaluations such as blood tests and imaging, and as an adjunctive marker for minimally invasively diagnosing malignant tumors alongside cytology for diagnosing malignant tumors. Cytological examination, commonly used in clinical practice, is a relatively simple diagnostic method for assessing malignancy, characterized by high specificity but low sensitivity. Therefore, the minimally invasive combination of serum PGRN with high sensitivity and cytologic evaluations with high specificity would be complementary in diagnosing malignancy. However, it is important to note that this study found no statistically significant difference in PGRN levels between malignant and benign tumor groups, highlighting the limited utility of PGRN alone for distinguishing malignancy.

Additionally, as a diagnostic biomarker, PGRN proved useful in distinguishing metastatic from non-metastatic tumors. PGRN levels were significantly higher in metastatic tumors compared to non-metastatic tumors (3.671 vs. 2.964 ng/mL, *p* = 0.0264). This was further corroborated by ROC analysis, which demonstrated the ability to differentiate metastatic status (AUC = 0.6514, *p* = 0.0129). According to previous study, PGRN-promoted migration and invasion have been closely associated with the activation of the epithelial–mesenchymal transition (EMT) process [28]. This process enables cells to leave the primary tumor by transforming stationary epithelial cells into mobile mesenchymal cells. Actually, the role of PGRN in promoting EMT has been extensively confirmed at the in vitro level across various cancer cell lines, including breast cancer MCF-7 cells, colorectal cancer SW1116 cells, and bladder cancer 5637 cells [29,30,31]. Therefore, it can be hypothesized that PGRN-mediated metastasis in dogs may similarly involve EMT mechanisms. Additionally, the observation in this study that PGRN levels were not significantly elevated in mesenchymal-origin tumors might indicate that EMT processes are less critical in these tumor types, although further studies are necessary to confirm this.

Based on clinical categorization, this study found that HLS tumors exhibited significantly elevated PGRN levels compared to the control group (3.733 vs. 2.054 ng/mL, *p* = 0.0007), demonstrating the highest diagnostic accuracy among the tumor subtypes (AUC = 0.835). These findings suggest that PGRN may serve as a biomarker for diagnosing multicentric lymphoma in dogs, which is consistent with previous studies in humans [23]. Although further studies comparing PGRN levels in dogs with generalized lymphadenopathy caused by other diseases versus multicentric lymphoma are needed, our results highlight PGRN’s potential as a useful serological biomarker for lymphoma diagnosis. Additionally, MGTs were found to have significantly higher PGRN levels compared to the control group (3.125 vs. 2.054 ng/mL, *p* = 0.0027). This finding aligns with previous research, where elevated serum PGRN levels were reported in breast cancer patients compared to healthy individuals [32], confirming similar observations in dogs in this study. For epithelial, mesenchymal, and neuroendocrine tumors, the Kruskal–Wallis test results showed no significant difference in serum PGRN levels compared to the control group. Due to the lack of clinical studies measuring serum PGRN in patients with these tumor types, direct comparisons remain challenging. However, the Mann–Whitney test results confirmed that dogs with epithelial tumors also had significantly higher serum PGRN levels compared to the control group. Although there are very limited findings in canine research, the results of this study are consistent with prior in vitro findings, which suggested that PGRN promotes cell proliferation in human epithelial cancer cell lines via the activation of the MAPK/ERK 1/2 and PI3K/AKT/mTOR signaling pathways [33,34,35,36,37,38,39].

This study has several limitations. First, due to the retrospective feature of this research, CT imaging was not performed for all dogs in the tumor group. Consequently, early-stage or micro-level metastases might have been missed, potentially affecting the accuracy of group classification based on metastatic status. Second, as a survival analysis through longitudinal follow-up was not conducted, the evaluation relied on indirect evidence inferred from malignancy and metastatic status, rather than definitive survival outcomes. Third, during the retrospective analysis of histopathological records, the results for 11 cases of MGTs were unavailable, and the availability of histopathological staging information was limited. Fourth, due to the retrospective nature of this study, complete demographic equivalence among the groups could not be ensured. Therefore, factors such as age, sex, neuter status, and breed might have influenced serum PGRN concentrations. In the same context, the neuroendocrine tumor group included only a small number of cases, which may have resulted in limited statistical power. Fifth, as the control group was retrospectively selected and follow-up was not conducted, the presence of subclinical neoplasms at the time of serum collection cannot be entirely ruled out. Finally, this study has inherent limitations regarding the variability of tumor characteristics and should be considered preliminary work requiring further validation.

## 5. Conclusions

This study demonstrated that PGRN holds significant potential as a serum biomarker with both diagnostic and prognostic relevance in the assessment of neoplastic conditions in dogs. Specifically, PGRN demonstrated notable sensitivity for tumor diagnosis, suggesting its possible utility as a screening tool. Furthermore, its diagnostic value in differentiating metastatic from non-metastatic tumors was evident. In addition, elevated PGRN levels were associated with malignant and metastatic tumors, which are generally linked to poor clinical outcomes. Based on these findings, PGRN may serve as a prognostic biomarker for identifying tumors with unfavorable biological characteristics. Overall, the results support the potential utility of PGRN as a serological marker for both early diagnostic and prognostic determination in canine neoplastic diseases.

## Figures and Tables

**Table 1:** Tumor types of 104 dogs based on practical clinical categorization in this study.

Signalment	Control		Tumor Types							
		MGT	HLS		Mesenchymal		Epithelial		Neuroendocrine	
			Benign	Malignant	Benign	Malignant	Benign	Malignant	Benign	Malignant
Number	30	45	0	20	3	13	4	15	1	3
Age (median [IQR])	8 [4–12]	10 [9–12]	-	8 [6–10.25]	9 [7.5–10]	11 [8–12]	10 [9.75–11]	11 [7–14.5]	13 [13]	10 [9.5–10.5]
Sex	F (0), SF (12), M (1), MN (15)	F (27), SF (18), M (0), MN (0)	-	F (4), SF (5), M (3), MN (8)	F (0), SF (1), M (0), MN (2)	F (1), SF (7), M (2), MN (3)	F (0), SF (1), M (2), MN (1)	F (0), SF (5), M (3), MN (7)	F (0), SF (1), M (0), MN (0)	F (0), SF (0), M (0), MN (3)
Breed	Beagle (1) Bichon Frise (4) Chihuahua (2) French Bulldog (2) Maltese (8) Mixed (7) Pomeranian (2) Poodle (2) Shih Tzu (1) Yorkshire Terrier (1)	Border Collie (1) Boston Terrier (1) Cocker Spaniel (2) Dachshund (1) Golden Retriever (1) Jindo Dog (2) Maltese (11) Miniature Pinscher (1) Mixed (9) Pomeranian (1) Poodle (8) Shih Tzu (5) Yorkshire Terrier (2)	-	Bichon Frise (1) Chihuahua (1) Cocker Spaniel (1) Maltese (3) Miniature Pinscher (1) Pompitz (2) Poodle (4) Shih Tzu (4) Yorkshire Terrier (3)	Bichon Frise (1) Mixed (1) Poodle (1)	Boston Terrier (3) Golden Retriever (2) Maltese (1) Miniature Schnauzer (2) Pomeranian (2) Schnauzer (1) Shih Tzu (1) Welsh Corgi (1)	Chihuahua (1) Doberman Pinscher (1) Maltese (1) Poodle (1)	Jindo Dog (2) Maltese (8) Mixed (1) Pompitz (2) Schnauzer (1) Shih Tzu (1)	Maltese (1)	Pomeranian (3)

IQR, interquartile range; F, female; SF, spayed female; M, male; MN, male neutered; MGT, mammary gland tumor; HLS, hematopoietic and lymphoreticular system.

**Table 2:** Practical clinical categorization of 104 canine tumors based on the definitive diagnosis.

**Mammary gland tumors (*n* = 45)**
• Tubulopapillary carcinoma (*n* = 8) • Complex carcinoma (*n* = 7) • Tubular carcinoma (*n* = 6) • Simple adenoma (*n* = 2) • Simple carcinoma (*n* = 1) • Solid carcinoma (*n* = 1) • In-situ carcinoma (*n* = 1) • Inflammatory carcinoma (*n* = 1) • Ductal carcinoma (*n* = 1) • The spindle cell variant of carcinoma (*n* = 1) • Intraductal papillary carcinoma (*n* = 1) • Intraductal papillary adenoma (*n* = 1) • Malignant myoepithelioma (*n* = 1) • Carcinoma and malignant myoepithelioma (*n* = 1) • Osteoblastic osteosarcoma (*n* = 1) • Unavailable (*n* = 11)
**Hematopoietic and lymphoreticular tumors (*n* = 20)**
Malignant (*n* = 20)
• Lymphoma (*n* = 16) • Mast cell tumor (*n* = 4)
**Mesenchymal (*n* = 16)**
Benign (*n* = 3)
• Hemangioma (*n* = 1) • Lipoma (*n* = 1) • Myolipoma (*n* = 1)
Malignant (*n* = 13)
• Osteosarcoma (*n* = 3) • Hemangiosarcoma (*n* = 4) • Melanoma (*n* = 4) • Soft tissue sarcoma (*n* = 1) • Cystic myxosarcoma (*n* = 1)
**Epithelial (*n* = 19)**
Benign (*n* = 4)
• Apocrine gland adenoma (*n* = 2) • Hyperplastic polyp (*n* = 1) • Renal cystic adenoma (*n* = 1)
Malignant (*n* = 15)
• Hepatocellular carcinoma (*n* = 5) • Adenocarcinoma (*n* = 5) • Squamous cell carcinoma (*n* = 3) • Renal cell carcinoma (*n* = 2)
**Neuroendocrine (*n* = 4)**
Benign (*n* = 1)
• Chemodectoma (*n* = 1)
Malignant (*n* = 3)
• Pheochromocytoma (*n* = 3)
**Total number of canine tumors (*n* = 104)**

**Table 3:** The results of ROC analyses with sensitivities and specificities based on the best cutoff value.

**Subjects of ROC Analysis** **(Best Cutoff Value)**	**AUC**	**Sensitivity (%)**	**Specificity (%)**	***p* Value**
Tumor vs. control (1.956 ng/mL)	0.7229	90.91%	50%	0.0002
MGT vs. control (1.956 ng/mL)	0.7337	97.78%	50%	0.0006
HLS tumor vs. control (3.224 ng/mL)	0.835	75%	80%	<0.0001
Metastasis vs. no-metastasis (3.248 ng/mL)	0.6514	71.88%	64.1%	0.0129

ROC, receiver operating characteristic; AUC, area under the curve; MGT, mammary gland tumor; HLS, hematopoietic and lymphoreticular system.

## Data Availability

The original contributions presented in this study are included in the article. Further inquiries can be directed to the corresponding author(s).

## Abbreviations

The following abbreviations are used in this manuscript:

ANOVA	Analysis of variance
AUC	Area under curve
ELISA	Enzyme-linked immunosorbent assay
FNA	Fine needle aspiration
GP88	88-kDa glycoprotein
HLS	Hematopoietic and lymphoreticular system
HRP	Horseradish peroxidase
MGT	Mammary gland tumor
PARR	Polymerase Chain Reaction for Antigen Receptor Rearrangement
PGRN	Progranulin
ROC	Receiver operating characteristic
